# Temporal associations between microclimate, adult *Aedes* mosquito indices, and dengue cases at the residence level in Malaysia: Implications for targeted interventions

**DOI:** 10.1371/journal.pone.0316564

**Published:** 2025-02-03

**Authors:** Nur Athen Mohd Hardy Abdullah, Nazri Che Dom, Biswajeet Pradhan, Siti Aekball Salleh, Rahmat Dapari

**Affiliations:** 1 Faculty of Health Sciences, Universiti Teknologi MARA (UiTM), UITM Cawangan Selangor, Puncak Alam, Selangor, Malaysia; 2 Integrated Mosquito Research Group (I-MeRGe), Universiti Teknologi MARA (UiTM), Puncak Alam, Selangor, Malaysia; 3 Institute for Biodiversity and Sustainable Development (IBSD), Universiti Teknologi MARA, Selangor, Malaysia; 4 Faculty of Medicine and Health Sciences, Integrated Dengue Research and Development, Universiti Putra Malaysia, Serdang, Selangor, Malaysia; 5 Centre for Advanced Modelling and Geospatial Information Systems (CAMGIS), School of Civil and Environmental Engineering, University of Technology Sydney, Ultimo, Australia; 6 Institute of Climate Change, Earth Observation Centre, Universiti Kebangsaan Malaysia, Bangi, Selangor, Malaysia; 7 Faculty of Medicine and Health Sciences, Department of Community Health, Universiti Putra Malaysia, Serdang, Selangor, Malaysia; World Health Organization, Regional Office for South-East Asia, INDIA

## Abstract

**Introduction:**

Dengue continues to be a major public health concern in Malaysia, as evidenced by the significant surge in cumulative dengue case numbers and deaths in 2023 compared to the previous year. While previous studies have explored the interplay of abiotic and biotic factors of mosquito density and dengue cases on a local scale in Malaysia, there is a notable gap in the research focusing on adult *Aedes* mosquito populations.

**Aims:**

This study aims to contribute to the existing knowledge by investigating the association and time lags (TLs) between daily microclimate (DM), mosquito indices (MIs), and dengue cases at the residence level.

**Methods:**

In this longitudinal study, field data were collected over 26 weeks using data loggers, gravid oviposit sticky (GOS) traps, and non-structural 1 (NS1) test kits in both non-dengue hotspot (NDH) and dengue hotspots (DH). The collected data encompassed DM variables, vegetation cover (VC), MIs, and number of dengue cases. An autocorrelation analysis was conducted to determine the TLs between MIs and their preceding values, while a cross-correlation analysis revealed the TLs between MIs and DM variables.

**Results:**

The study indicated there are positive correlations between the adult index (AI) of *Ae*. *albopictus*, their preceding values and rainfall at an NDH. Conversely, the AIs of total *Aedes* at the DH exhibited positive correlations with their preceding values, temperature, rainfall, and maximum relative humidity (RH), but negative correlations with the mean and maximum RH. The dengue-positive trap index (DPTI) of total *Aedes* at DHs demonstrated positive associations with their preceding values, mean temperature, minimum temperature, maximum RH, and rainfall, with negative correlations observed for the maximum temperature, mean RH, and minimum RH. Similar trends were identified for the *Ae*. *aegypti* and *Ae*. *albopictus* at DHs. The association between dengue cases, DM, and MIs was inconclusive due to underreported cases.

**Conclusions:**

This study highlighted the DM and TLs of dengue virus-infected and non-infected adult female *Aedes* mosquitoes using onsite data collection. Furthermore, this study presents a replicable methodology that can be adopted by researchers worldwide for investigating the dynamics of dengue transmission in similar settings. The findings offer valuable insights for decision-makers, providing them with evidence-based information to implement targeted interventions and strategies aimed at controlling *Aedes* mosquito populations and mitigating the spread of dengue virus infections.

## Introduction

Dengue is a viral disease transmitted by mosquitoes, is caused by four closely related serotypes of the dengue virus [1]. In Malaysia, the primary vector for this disease is the *Ae*. *aegypti*, while the *Ae*. *albopictus* serves as a secondary vector [2]. The dengue virus is transmitted vertically through the bite of an infected mosquito [3,4]. Both the *Ae*. *aegypti* and *Ae*. *albopictus* are known to coexist in urban and suburban areas in Malaysia, and they are considered to be sympatric species occupying similar ecological niches [5–9]. The surveillance and control of *Aedes* mosquitoes remain a high priority, given the high dengue case numbers and mortality rates in Malaysia. As of 2023, the cumulative dengue cases reported was 123133, with 100 deaths. These figures represent an alarming increase of 86.3% in cumulative cases and 78.6% in deaths compared to the previous year [10].

The five strategies implemented in Integrated Vector Management for dengue in Malaysia includes reprioritising *Aedes* surveillance areas, strengthening information system for effective disease surveillance and response, legislative changes, community participation and intersectoral collaboration, as well as changing fogging formulation to water-based pyrethroid and mass abating [11]. In the event of a new dengue case or controlled outbreak, the local authorities are required to conduct the destruction of mosquito breeding places and fogging within 200 metres of case in 24 hours [12]. Meanwhile, local authorities are required to conduct these activities within 400 metres of case in 24 hours for uncontrolled outbreak and hotspot area [12]. Most of the dengue prevention measures are passive, as they can only be conducted after dengue cases are registered in the eDengue database. The dengue carrier needs to seek treatment and meet the clinical case definition and laboratory confirmation of dengue fever before being registered in eDengue [13]. This delay may cause the effective time for prevention measures to elapse.

Prior studies have pointed out the importance of climate factors, especially temperature, humidity, and rainfall, on dengue transmission as they are crucial to the mosquito population, mosquito density, and mosquito survival rate [14–16]. For instance, ambient temperatures can alter the lifespan of the mosquito and the extrinsic incubation period of the dengue virus. In turn, these traits affect the rate of pathogen transmission [17,18]. Meanwhile, RH can cause mortality in adult mosquitoes at low humidity, whereas high RH is associated with heavy rainfall [19–21]. Rainfall increases the mosquito population by generating mosquito breeding sites and vegetation cover (VC) [22]. The cross-correlation between rainfall and dengue increases, especially in cities with adequate water sanitation, such as piped water and sewerage [23]. At the same time, heavy rainfall decreases the mosquito density by flushing away those mosquitoes in their immature life stages [23]. Therefore, total rainfall one month prior has the most significant association with both the number of dengue cases and mosquito density [24,25].

Although, on a large topographical scale, the spread of mosquito vectors is mainly affected by climate, the *Aedes* mosquito population is influenced by the availability of larval habitats, VC, and daily microclimate (DM) on a local scale [26–29]. Therefore, the association of abiotic and biotic factors to mosquito density, and at sites is well established in Malaysia [30–35]. However, there are very few studies on adult *Aedes* mosquito populations as most researchers prefer to use the ovitrap index and number of larvae as the mosquito density variables [32,33]. However, immature stage indicators do not directly correlate with the risk of dengue infection and do not always spatiotemporally correlate with adult indicators [36–38].

Additionally, many studies on adult *Aedes* mosquitoes in Malaysia are aimed at finding the most efficient adult mosquito traps and use the non-structural 1 (NS1) antigen test kit on pooled trapped adult mosquitoes as an early dengue surveillance or control [39–42]. According to their research, adult *Aedes* mosquitoes can be effectively captured using sticky ovitraps, and the NS1 test kits can successfully detect the dengue virus in the captured adult mosquitoes. Therefore, this study was aimed at expanding the knowledge on the correlation and time lags (TLs) between different DMs, adult female mosquito indices (MIs), and dengue cases at both a dengue hotspot (DH) and non-dengue hotspot (NDH). This study utilised gravid oviposit sticky (GOS) traps and NS1 test kits to detect mosquito density and the presence of the dengue virus in a sample of female adult *Aedes* mosquitoes.

## Methods

### Study sites

A longitudinal study was conducted at two hostels, namely, a NDH and a DH. The NDH was located in Jeram, a sub-district in Kuala Selangor. It was selected because it was not recorded as a DH for the period 2017–2021. The NDH had four 10-storey blocks that accommodated 3003 people. It was selected as the control site after a preliminary study was conducted using ovitraps and larvae surveys detect the presence of *Ae*. *albopictus* larvae in ovitraps, drains, and vases in the area. The preliminary study showed that this residential environment was suitable for the survival and development of *Aedes* mosquitoes. On the other hand, the DH, which was located in Bukit Raja, a sub-district in Petaling, had been identified as such in 2017, 2018, and 2020. The DH had five 4-storey blocks accommodating 2870 people. These sites were selected because the population in both study sites spends a significant amount of time within the area, reducing the risk of dengue transmission from neighbouring workplaces. Additionally, both sites have their own dengue preventive measures which include monthly fogging, and this preventive measure continues during data collection. The data collection was conducted from 6^th^ February until 6^th^ August 2023 (26 weeks) with permission from local authority.

### Dengue case

The data on dengue cases at the study sites were taken from the Idengue website, the information in this system is obtained from Ministry of Health Malaysia and updated daily.

### Field data collection

#### Daily microclimate (DM)

The DM parameters collected at both study sites were the RH, rainfall, and temperature. The RH and temperature parameters were collected hourly using Tinytag Plus 2 (Gemini Data Loggers). The data loggers were placed in a well-ventilated area and mounted 2 metres above ground. Meanwhile, the rainfall data were collected from 11 rain gauge stations in the Kuala Selangor district and 15 stations in the Petaling district (S1 Table), which were updated daily on the Info Banjir JPS Selangor website [43].

#### *Aedes* mosquito collection

The *Aedes* mosquitoes were captured using GOS traps, comprising a black cylindrical high-density polyethylene (HDPE) plastic container (15 x 8 cm) and two double-sided sticky papers (5 x 3 cm). A mesh with a 4-cm hole at the centre was placed on the top of the entrance to the container to prevent larger insects from entering the trap. Then, the sticky papers were placed near the hole parallel to one another. The container was filled with 300 mL of 10% hay infusion water made from week-old hay as the attractant [44]. Furthermore, 20 GOS traps were placed 20 m apart from each other at each study site [45,46]. This spacing provided an even trap placement coverage, and *Aedes* mosquitoes were captured across the entire study site. The contents of the trap were collected weekly, and any missing or damaged traps were recorded and then replaced.

#### Vegetation cover (VC)

The onsite VC was visually assessed, as per the study by Walker et al. [47]. The values reflected the percentage of green VC area within a 3-m radius of the traps. The ‘per cent vegetation’ followed the protocols by Walker et al. [47], which involved an estimation by visual examination by a single viewer for consistency. The sites were classified into one of four VC levels: 1  = < 10% VC, 2 =  10–25% VC, 3 =  25–50% VC, and 4   =  >50% VC [47].

#### Detection of dengue non-structural 1 (NS1) antigen

The trapped adult *Aedes* mosquitoes were sorted and identified using a Dino-Lite handheld digital microscope (AnMo Electronics Corporation) and based on the morphological keys in the laboratory. Next, the female adult *Ae*. *aegypti* and *Ae*. *albopictus* mosquitoes were pooled per trap for the NS1 antigen test according to the procedures modified by Cheng et al. [48]. The mosquito sample was prepared by homogenising the *Aedes* mosquito pools in 1.5-mL tubes containing 200 μl of phosphate-buffered saline (PBS) using pellet pestles. The ProDetect® Dengue NS1 Ag Rapid Test was used for the NS1 antigen detection. The NS1 disposable pipette provided inside the kit was used to collect 75 μL of the sample, which was dropped into the sample well of the cassette. The result was recorded after 10 minutes, as stated in the manufacturer’s information.

### Data analysis

#### Daily microclimate (DM) description and interpolation

The hourly RH and temperature collected from the data logger were calculated into a daily and weekly format in Microsoft® Excel™ Open XML Spreadsheet (XLSX). Meanwhile, all the rain gauge stations were geocoded to produce a point shapefile using ArcGIS Desktop Version 10.8. This shapefile was used in the inverse distance weighting (IDW) interpolation to estimate the rainfall at the study sites based on the data at nearby rain gauge stations. This method was selected due to its high performance for univariate interpolations [49,50]. The IDW function in ArcGIS was used with default settings, with the power set to 2 to control the significance of known points upon the interpolated values. The IDW was computed as:

$$w(x,y)=\sum_{i=1}^{N}\lambda_{i}w_{i}\lambda_{i}=\frac{d_{i}^{-p}}{\sum_{i=1}^{N}d_{k}^{-p}}$$ (1)

where: *w*(*x*, *y*) is the estimated results at points (*x*, *y*), *N* is the number of observational points surrounding (*x*, *y*), *λ*_*i*_ is the weight of the observed results (*w*_*i*_) at points (*x*_*i*_, *y*_*i*_), *d*_*i*_ is the distance between points (*x*_*i*_, *y*_*i*_) and (*x*, *y*). The exponent (*p*) affects the weighting of *w*_*i*_ on *w* [46]. In this study, *p* = 2. The values on the maps that coincided with the study sites were then extracted and organised in Microsoft® Excel™ Open XLSX into daily and weekly formats.

#### Mosquito indices (MIs) and daily interpolation

Meanwhile, the outcomes of the field data collection were measured in terms of weekly MIs. These MIs were further segregated into total *Aedes*, *Ae*. *albopictus*, and *Ae*. *aegypti*. The indices were the adult sticky trap index (ASTI), adult index (AI), and dengue-positive trap index (DPTI). The indices, which were adapted and modified from Liew et al. [44], were calculated as follows:

$$ASTI=\left(\frac{Number\;of\;traps\;with\;adult\;female\;Aedes\;sp.}{Total\;number\;of\;inspected\;traps}\right)\times 100\%$$ (2)

$$AI=\left(\frac{Number\;of\;female\;adult\;Aedes\;sp.collected}{Total\;number\;of\;inspected\;traps}\right)\times 100\%$$ (3)

$$DPTI=\left(\frac{Number\;of\;traps\;with\;adult\;female\;Aedes\;sp.positive\;for\;dengue\;NS1}{Total\;number\;of\;inspected\;traps}\right)\times 100\%$$ (4)

Only AI and DPTI were used in the correlation analysis. These values were converted into daily data using cubic spline interpolation in R software. The interpolation provided a means of estimating the value at the new data points within the range of parameters, and the cubic spline interpolation formed a smooth curve through a series of shape points [51]. Taking (*n*+1) nodes on the interval [*a*, *b*]:

$$a=x_{0}<x_{1}<\cdots <x_{n}=b.$$ (5)

At each interval [*x*_*i*−1_, *x*_*i*_], *f*(*x*) is a cubic polynomial function,

$$f_{i}(x)=a_{i}+b_{i}(x_{i-1},x_{i})+{c_{i}(x_{i-1},x_{i})}^{2}+{d_{i}(x_{i-1},x_{i})}^{3},$$ (6)

where, *f*(*x*) is continuous in the interval [*a*, *b*].


$$f(x_{0})=y_{0},\ldots ,f(x_{n+1})=y_{n+1},$$ (7)

$$f_{-}=f_{+}{(x}_{i})=y_{i},i=1,2,\ldots ,n,$$ (8)

In this study, a cubic spline interpolation was used to interpolate at the starting point six path nodes and the target point to form a completely smooth spline by connecting all the interpolation points. The interpolated data were used for a correlation analysis.

#### Autocorrelation and cross-correlation

An autocorrelation analysis was conducted in R software to determine if the daily AI and DPTI and dengue cases were affected by their own preceding values with TLs of 1 to 91 days using the autocorrelation coefficient and partial autocorrelation coefficient. {*X*_*t*_} was a stationary time series with length *T*. A time series {*X*_*t*_} with a mean function, μ_*t*_ = *E*[*X*_*t*_] and autocovariance function [52]:

$$\gamma_{X}(h)=Cov(X_{t},X_{t-h})=E[(X_{t}-\mu_{X})(X_{t-h}-\mu_{X})]$$ (9)

where, {*X*_*t*−*h*_} is the lagged time series by *h* periods and μ_*X*_ is the expected value of {*X*_*t*_}. The autocorrelation function (ACF) was:

$$\rho_{X}(h)=\frac{\gamma_{X}(h)}{\gamma_{X}(0)}=Cor(X_{t},X_{t-h})$$ (10)

The partial autocorrelation function (PACF) measures the degree of association between {*X*_*t*_} and {*X*_*t*−*h*_}, whereas the other TLs were not considered. The PACF was calculated as:

$$\frac{Cov(X_{t}|X_{t-1},X_{t-2}|X_{t-2})}{\sqrt{Var(X_{t}/X_{t-1})Var(X_{t-2}/X_{t-1})}}$$ (11)

The cross-correlation analysis was conducted to determine the optimal TL between DM, AI, DPTI and dengue cases between 0 to 91 days. The cross-correlation function (CCF) between the variable {*X*_*i*_} and {*X*_*j*_} was defined by the ratio of covariance to root-mean variance [53]:

$$\rho_{i,j}=\frac{\gamma_{i,j}}{\sqrt{\sigma_{i}^{2}\sigma_{j}^{2}}}=\frac{\sum_{t=1}^{N}\left[\left(X_{i}^{t}-X_{i})(X_{j}^{t}-X_{j}\right)\right]}{\sqrt{\sum_{t=1}^{N}{\left(X_{i}-X_{i}\right)}^{2}\sum_{t=1}^{N}{\left(X_{j}-X_{j}\right)}^{2}}}$$ (12)

where, the sample covariance {*γ*_*i*,*j*_} was found using:

$$\gamma_{i,j}=\frac{1}{N}\sum_{t=1}^{N}\left[\left(X_{i}^{t}-X_{i})(X_{j}^{t}-X_{j}\right)\right]$$ (13)

The critical values for all the correlation analyses were set at the 5% significance level (*p* < 0.05). Lastly, the correlation analysis at the DH was further segregated into total *Aedes*, *Ae*. *albopictus*, and *Ae*. *aegypti*.

#### Ethics approval

This study received an Ethics Review Exemption—UiTM Research Ethics Committee REC/01/2023 (PG/EX/2), dated 16 January 2023.

## Results

### Daily microclimate (DM)

**Fig 1:**
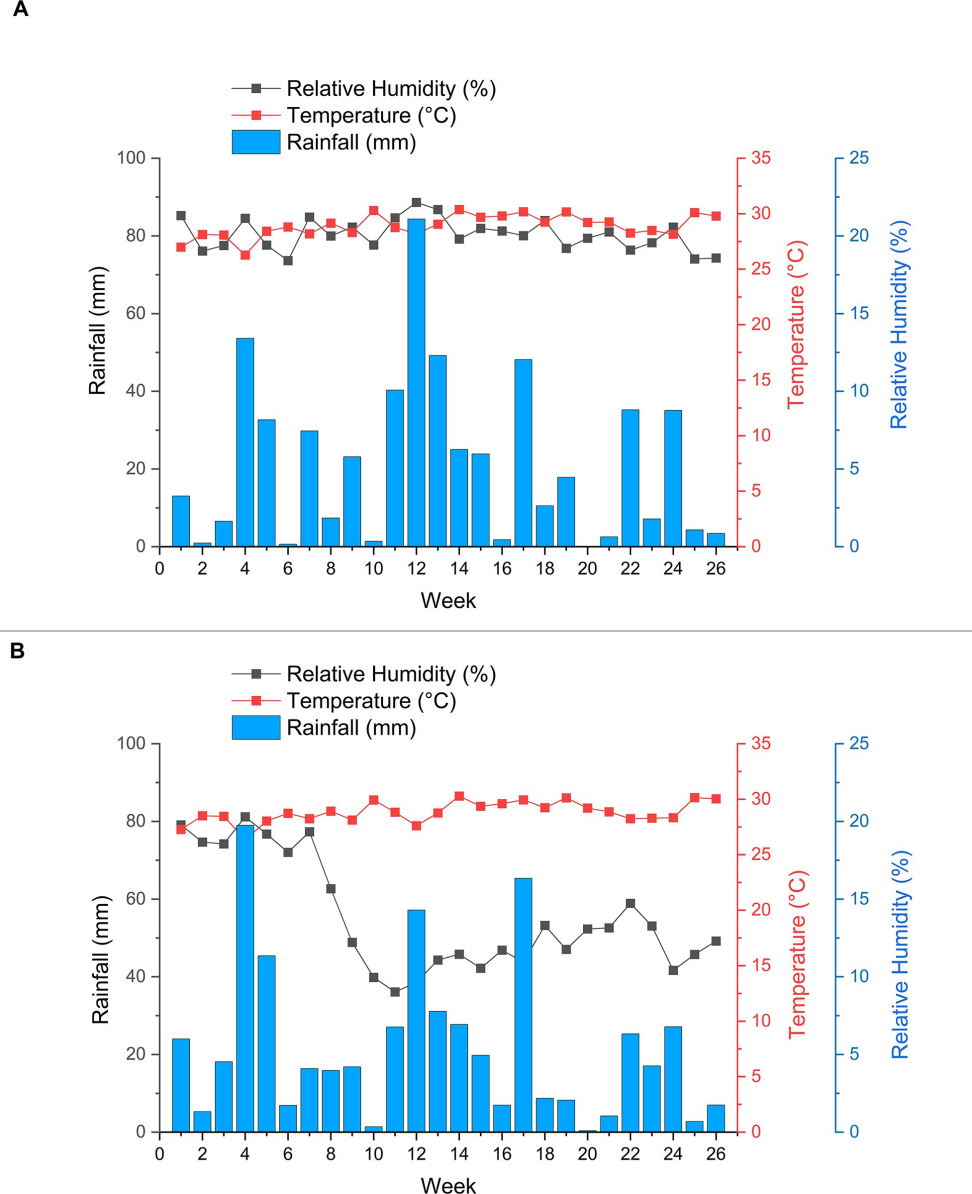
Weekly mean daily microclimate at the (A) NDH and (B) DH.

The daily mean temperature at the NDH during the study period was 28.90°C (SD = 1.33). The lowest and highest daily mean temperatures recorded were 24.29 and 31.62°C, respectively. Meanwhile, the daily mean RH was 80.33% (SD = 6.79), with 53.59% and 96.78% being the lowest and highest daily mean humidity recorded, respectively. The daily mean rainfall was 5.37 mm (SD = 10.62) and the highest rainfall recorded was 64.66 mm. Meanwhile, the weekly mean temperature and RH at the NDH indicated that the parameters remained constant during the study period. The mean temperature ranged from 26.28–30.38°C, while the mean humidity ranged from 73.6–88.63%. In contrast, there were several weeks when the mean weekly rainfall was high in NDH. The highest weekly mean rainfall recorded was in Week 12 with 21.09 mm (SD = 10.95). Other weeks with a high weekly mean rainfall were Week 4 (M = 13.40 mm, SD = 10.86), Week 13 (M = 12.31 mm, SD = 20.26), and Week 17 (M = 12.04 mm, SD = 23.65). Fig 1A below summarises the weekly mean DM at the NDH.

At the DH, the daily mean temperature during the study period was 28.83°C (SD = 1.21). The lowest daily mean temperature recorded was 24.80°C and the highest was 31.38°C. The daily mean RH was 55.31% (SD = 15.95), with 30.36 and 90.54% being the lowest and highest daily RH recorded, respectively. The daily mean rainfall was 5.37 mm (SD = 9.64) and the highest daily mean rainfall recorded was 79.19 mm. The weekly mean temperature at DH shows that the parameter remained constant during the study period. The mean temperature ranged from 26.37–30.28°C. On the other hand, the weekly mean humidity remained constant within 72.06–81.18% from Weeks 1–7. The humidity significantly decreased from 81.18% to 36.10% between Weeks 7–11 before gradually increasing and remaining within the range of 38.55–58.92% for the remainder of the study period. There were several weeks when the weekly mean rainfall at the DH was high. The highest mean rainfall recorded was in Week 4 with 19.75 mm (SD = 16.05). The other weeks with a high mean rainfall were Week 5 (M = 11.35 mm, SD = 10.92), Week 12 (M = 14.29 mm, SD = 10.94), and Week 17 (M = 16.34 mm, SD = 29.10). Fig 1B summarises the weekly mean DM at the DH.

### Vegetation cover (VC)

The GOS traps at the NDH had different VC levels. Most had < 10% of VC (70%), five had 10–25% of VC (25%), and only one had 25–50% of VC (5%). The NDH had different types of VC, which decreased as it got closer to the buildings at the site. In contrast, all the traps in the DH had < 10% of VC as the site was compacted with buildings and most of the area was asphalted, leaving only a small area for VC. Table 1 shows the VC within a 30-m radius of the ovitraps at each study site.

**Table 1:** The vegetation cover (VC) level at the NDH and DH.

No.	NDH			DH		
	GOS trap ID	VC level	VC level interpretation	GOS trap ID	VC level	VC level interpretation
**1**	1	3	25 to 50% coverage	21	1	<10% coverage
**2**	2	2	10 to 25% coverage	22	1	<10% coverage
**3**	3	2	10 to 25% coverage	23	1	<10% coverage
**4**	4	2	10 to 25% coverage	24	1	<10% coverage
**5**	5	1	<10% coverage	25	1	<10% coverage
**6**	6	1	<10% coverage	26	1	<10% coverage
**7**	7	1	<10% coverage	27	1	<10% coverage
**8**	8	1	<10% coverage	28	1	<10% coverage
**9**	9	1	<10% coverage	29	1	<10% coverage
**10**	10	1	<10% coverage	30	1	<10% coverage
**11**	11	1	<10% coverage	31	1	<10% coverage
**12**	12	2	10 to 25% coverage	32	1	<10% coverage
**13**	13	1	<10% coverage	33	1	<10% coverage
**14**	14	1	<10% coverage	34	1	<10% coverage
**15**	15	1	<10% coverage	35	1	<10% coverage
**16**	16	1	<10% coverage	36	1	<10% coverage
**17**	17	2	10 to 25% coverage	37	1	<10% coverage
**18**	18	1	<10% coverage	38	1	<10% coverage
**19**	19	1	<10% coverage	39	1	<10% coverage
**20**	20	1	<10% coverage	40	1	<10% coverage

Note: VC refer to vegetation cover; NDH: Non dengue hotspot area and DH: Dengue hotspot.

### Mosquito Indices (MIs)

A total of 529 adult female *Ae*. *albopictus* mosquitoes were trapped at the NDH with a capture rate per week of 20.0 adult gravid female mosquitoes (SD = 6.44). No adult female *Ae*. *aegypti* were captured in NDH. There were no mixed captured containers and dengue cases at that site. The mean ASTI of *Ae*. *albopictus* in the NDH was 57.74% trap per week. Moreover, the mean AI of *Ae*. *albopictus* during the study period was 103.15%, which suggested that there was a high female adult population in the surveyed area even with continuous dengue interventions conducted at NDH. Meanwhile, *Ae*. *albopictus* (258 adult females) and *Ae*. *aegypti* (156 adult females) at the DH had a mean capture rate of 9.92 (SD = 5.20) and 6.00 (SD = 3.96) mosquitoes per week, respectively. Additionally, the rate for both species captured in the same container was 1.88 (SD = 1.68) traps per week, while the rate of dengue cases was 0.07 (SD = 0.09) case per week. The mean ASTI for the *Ae*. *albopictus* was 36.30%, while for the *Ae*. *aegypti* it was 19.87%. Meanwhile, the mean AI for *Ae*. *albopictus* and *Ae*. *aegypti* was 50.14% and 30.46%, respectively. This suggested that there was a moderate adult female population for both *Aedes* species at the DH, even though there were continuous dengue interventions during study period. Moreover, the mean DPTI was 1.93% for *Ae*. *albopictus* and 2.54% for *Ae*. *aegypti*. Table 2 summarises the weekly mean mosquito captured rate and MIs at the study sites.

**Table 2:** Weekly mean of capture rate and MIs at the NDH and DH.

**No.**	**Study site**	**Species**	**Female mosquitoes captured** **(SD)**	**ASTI (%)**	**AI (%)**	**DPTI (%)**
**1**	NDH	*Ae*. *albopictus*	20.0 (6.44)	57.74	103.15	0.00
		*Ae*. *aegypti*	-	-	-	-
**2**	DH	*Ae*. *albopictus*	9.92 (5.20)	36.30	50.14	1.93
		*Ae*. *aegypti*	6.00 (3.96)	19.87	30.46	2.54

Note: The indices are adult sticky trap index (ASTI), adult index (AI), and dengue positive trap index (DPTI).

**Fig 2:**
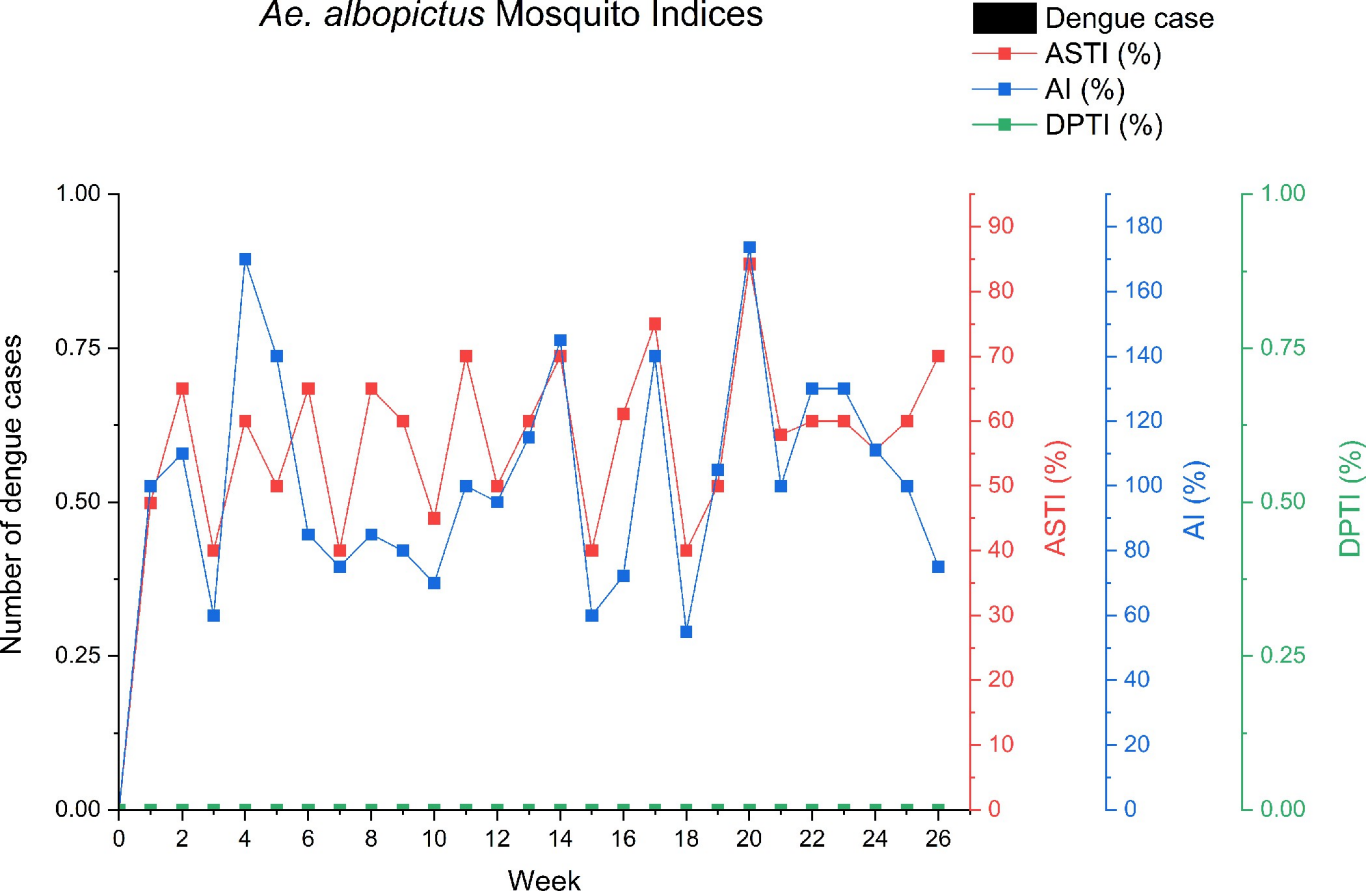
Weekly pattern of mosquito indices at the NDH based on adult sticky trap index (ASTI), adult index (AI), and dengue-positive trap index (DPTI).

A weekly MIs analysis was conducted at the NDH and DH. For the NDH, the range of weekly ASTI of *Ae*. *albopictus* was 40.00–84.21%. The highest weekly ASTI for *Ae*. *albopictus* was in Week 20 (%). The lowest weekly ASTI was in Weeks 3, 15, and 18. Other than that, AIs of *Ae*. *albopictus* greater than 100% were detected in Weeks 2 (110.0%), 4 (170.00%), 5 (140.00%), 17 (140.00%), 19 (100.50%), 20 (173.68%), 23 (130.00%), 24 (111.11%), and 25 (100.00%). Although there was a high AI at the NDH, the NS1 test indicated that there was no dengue virus detected in the captured *Aedes* mosquitoes. This was also reflected by the zero dengue case reported at the site throughout the study period. Fig 2 shows the weekly MIs at the NDH.

For the *Ae*. *albopictus* at DH, the highest ASTI was in Weeks 14 and 24 (60.00%). On the other hand, the lowest ASTI was in Week 4 (15.00%). There were several weeks when the AI was high for this mosquito species. These included Weeks 1(80.00%), 14 (100.00%), and 24 (100.00%). The DPTI of the *Ae*. *albopictus* was constant between 5 to 10% during the study period.

**Fig 3:**
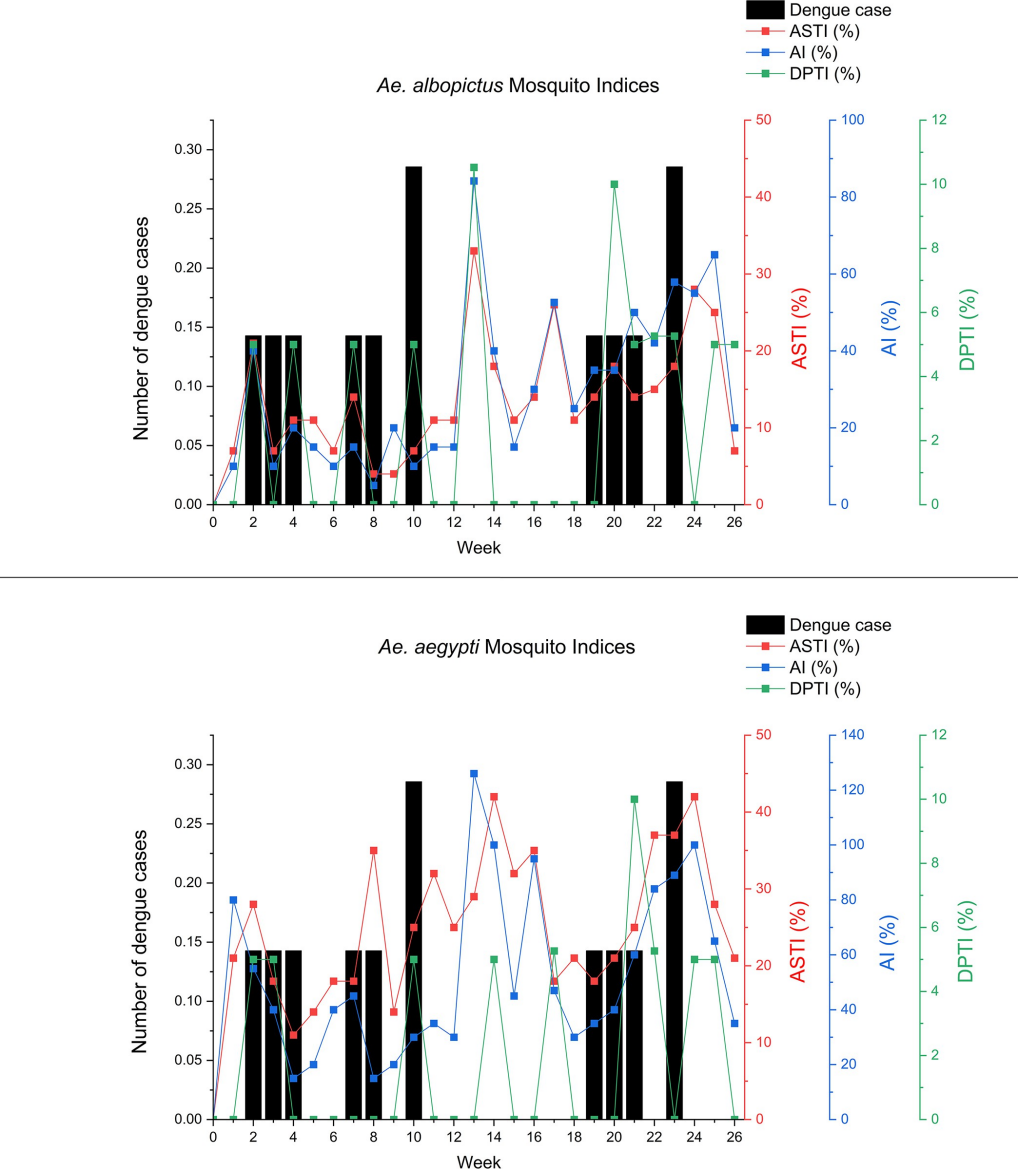
Species pattern of *Ae*. *albopictus* and *Ae*. *aegypti* at the DH based on adult sticky trap index (ASTI), adult index (AI), and dengue-positive trap index (DPTI).

Meanwhile, the weekly ASTI for *Ae*. *aegypti* at the DH was 5.00–47.37%, with the highest ASTI being in Week 13 and the lowest in Week 8. Other weeks with a high ASTI were Weeks 17, 24 and 25, with 36.84%, 40.00% and 35.00% respectively. Moreover, a few high AIs for *Ae*. *aegypti* were identified in Weeks 13 (84.21%), 17 (52.63%), 23 (57.89%), and 25 (65.00%). Lastly, the weekly DPTI for *Ae*. *aegypti* was 5.0–10.5%. Fig 3 illustrates the weekly MIs for the *Ae*. *albopictus* and *Ae*. *aegypti* at the DH.

#### Correlation between daily microclimate (DM) and mosquito indices (MIs)

**Fig 4:**
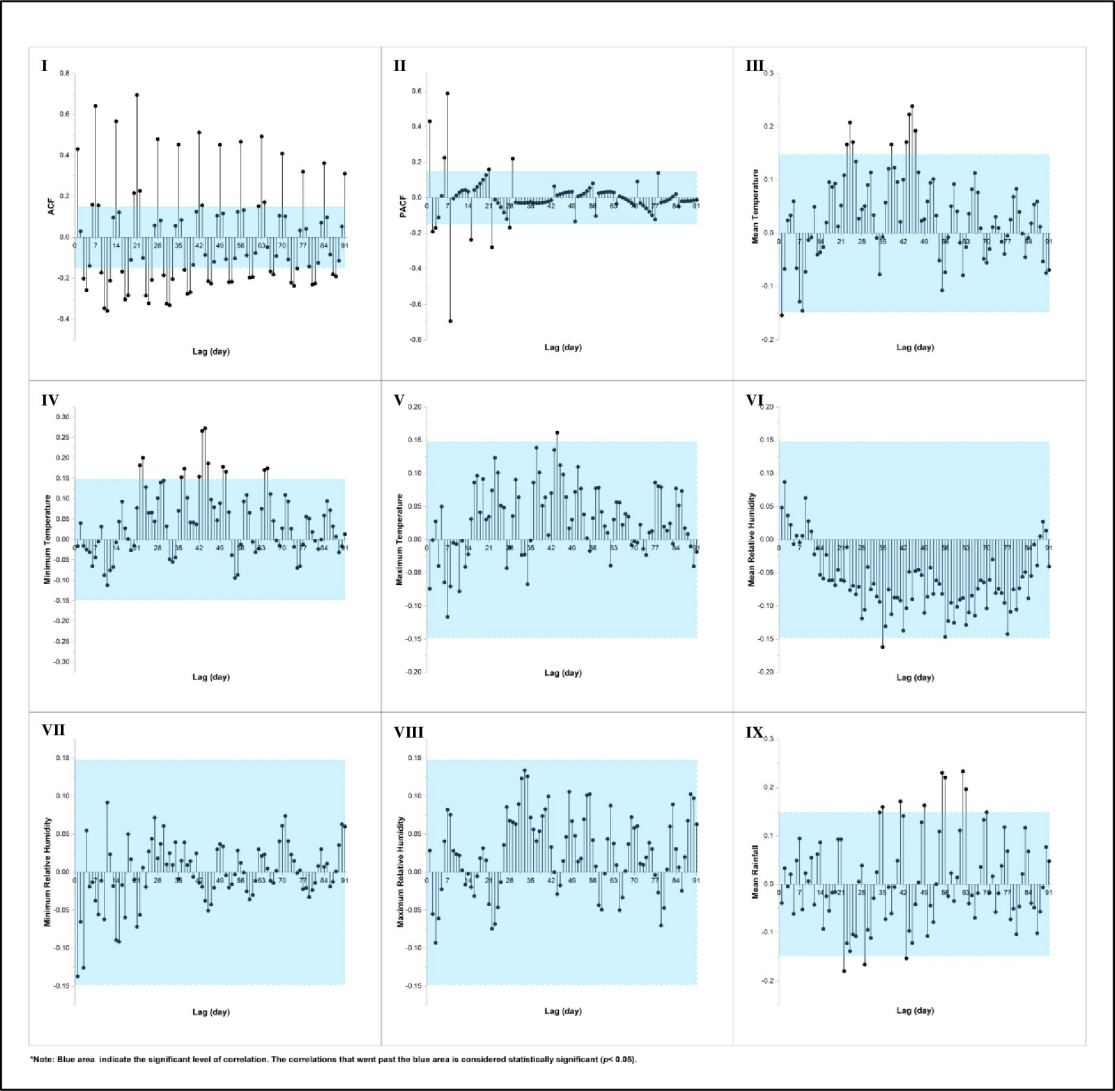
Autocorrelation (I and II) and cross-correlation (III to IX) between adult index and daily microclimate at the NDH area over 91 lag days. Note: Blue areas indicate significant levels of correlation.

The autocorrelation analysis of the AI for *Ae*. *albopictus* at the NDH indicated that the optimal correlation of AI lags was on Day 21 (*r* = 0.70). On the other hand, the PACF result showed that the optimal correlation was on Day 8 (*r* = -0.70). The cross-correlation analysis between the daily AI and DM at the NDH showed that the optimum TL for the mean temperature was on Lag Day 45 (*r* = 0.24), while the optimum TL for the minimum (*r* = 0.27) and maximum temperature (*r* = 0.16) was on Day 44. Additionally, the optimum lag for the mean RH and rainfall was on Day 35 (*r* = -0.16) and 62 (*r* = 0.23), respectively. Lastly, the analysis indicated that there was no significant correlation between the AI and the minimum and maximum RH. The correlation plots for the AI and DM of the NDH are displayed in Fig 4.

The analysis of the ACF and PACF of the AI for the total *Aedes* species and its preceding values at the DH revealed that the optimum TLs occurred on Lag Days 7 (*r* = 0.69) and 1 (*r* = 0.62). Additionally, the optimal lag for the mean temperature, minimum temperature, and maximum temperature was observed on Lag Day 52 (*r* = 0.32), 43 (*r* = 0.29), and 51 (*r* = 0.23), respectively. The optimum lag for the mean RH was on Day 26 (*r* = -0.37), while for the minimum and maximum RH, it was on Lag Day 28 (*r* = -0.42 and *r* = 0.26, respectively). Lastly, the optimal lag for rainfall was identified on Day 84 (*r* = 0.20).

**Fig 5:**
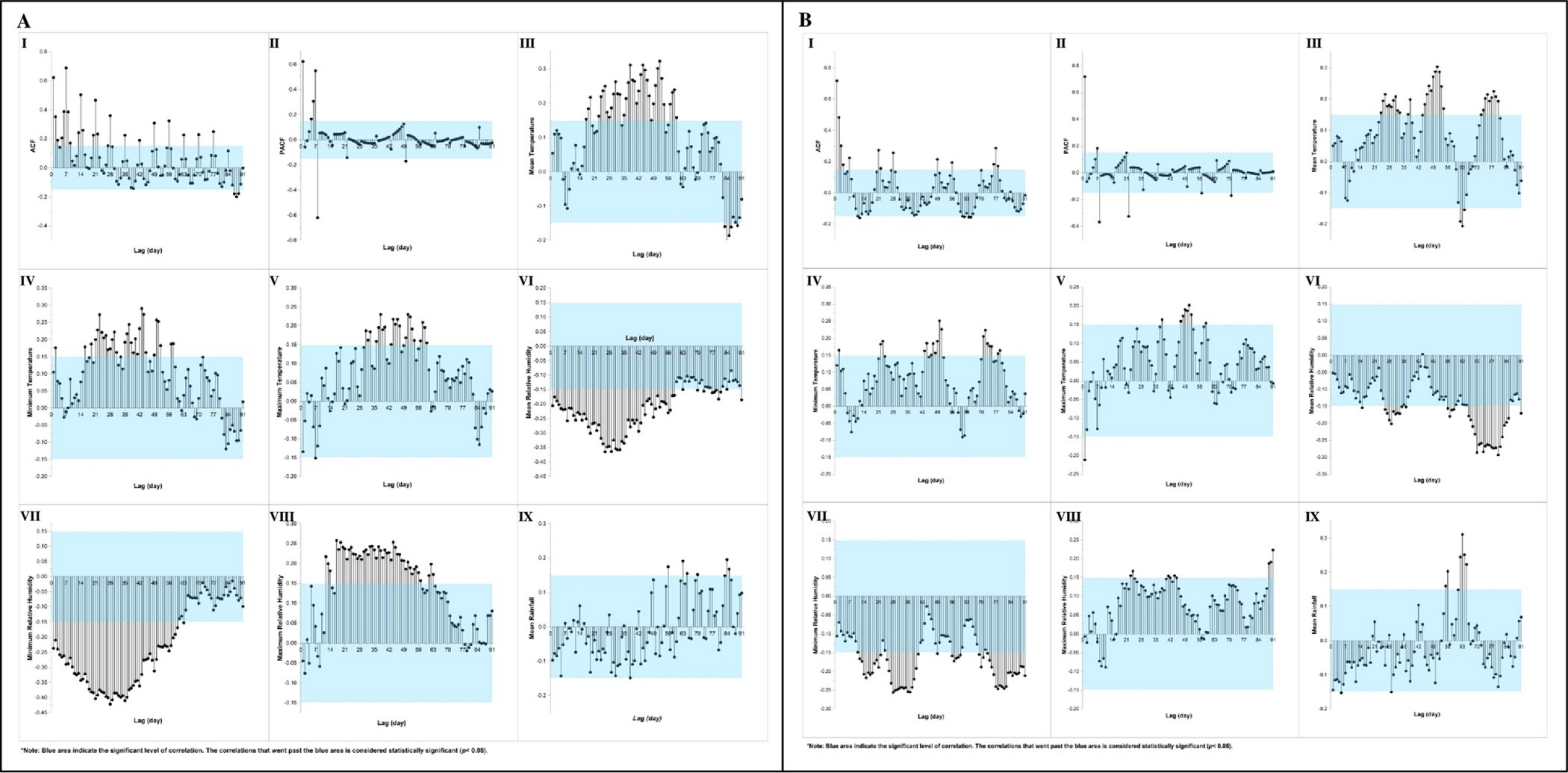
Autocorrelation (I and II) and cross-correlation (III to IX) of (A) adult index (AI), and (B) dengue positive trap index (DPTI) of total *Aedes* species on daily microclimate at DH area over 91 lag days. Note: Blue areas indicate significant levels of correlation.

Meanwhile, the ACF and PACF between the DPTI of the total *Aedes* species and its prior values identified the optimum TL as being on Lag Day 1 (*r* = 0.72). The cross-correlation analysis showed that the optimum lag for the mean, minimum, and maximum temperature was on Lag Day 51 (*r* = 0.30), 50 (*r* = 0.25), and 1 (*r* = 0.20), respectively. Moreover, the optimum lag for the mean, minimum, and maximum RH was on Day 80 (*r* = -0.29), 28 (*r* = -0.26), and 91 (*r* = 0.22), respectively. For the rainfall, the optimum lag was on Day 64 (*r* = 0.31). Fig 5 shows the correlation plots between the mosquito density and daily microclimate variables of the *Aedes* at the NDH.

A further analysis was conducted to determine the correlation between the DM and MIs by species at the DH. The autocorrelation analysis detected the optimum lag of the AI of the *Ae*. *Aegypti* to its prior values was on Lag Day 1 (*r =* 0.64). The cross-correlation analysis between the AI and DM showed that the optimum lag for the mean, minimum, and maximum temperature was on Day 24 (*r =* 0.32), 23 (*r =* 0.33), and 38 (*r =* 0.25), respectively. The optimum lag for the mean, minimum, and maximum RH was on Day 26 (*r =* - 0.39), 28 (*r =* - 0.44), and 37 (*r =* 0.27), respectively. Lastly, the optimum lag for the rainfall was on Day 56 (*r =* 0.25).

**Fig 6:**
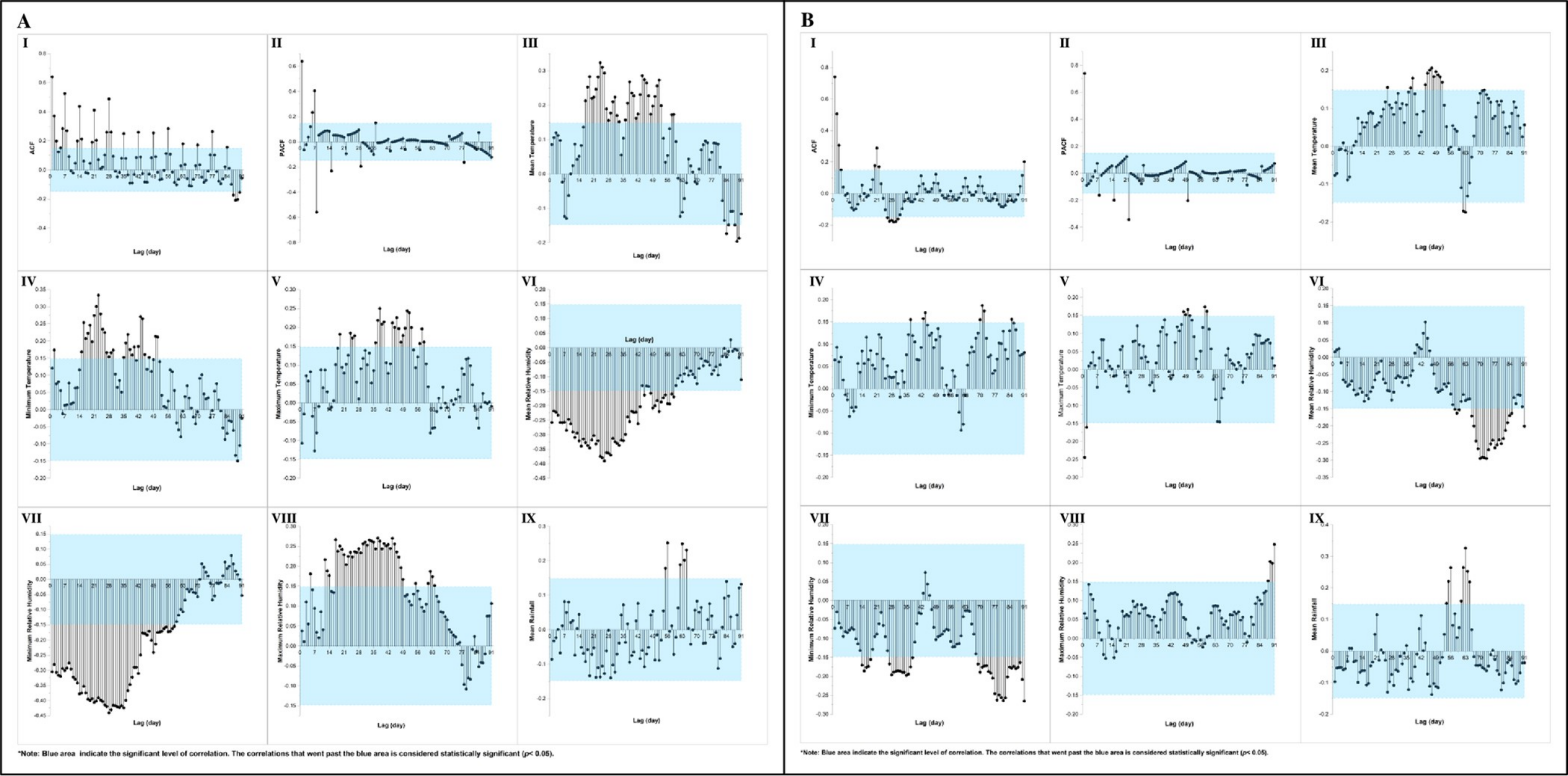
Autocorrelation (I and II) and cross-correlation (III to IX) of *Ae*. *aegypti* based on (A) adult index (AI), and (B) dengue positive trap index (DPTI) on daily microclimate at DH over 91 lag days. Note: Blue areas indicate significant levels of correlation.

The autocorrelation analysis between the DPTI of the *Ae*. *aegypti* with its earlier values implied that the optimum lag was on Lag Day 1 (*r =* 0.74). Furthermore, the cross-correlation between the DPTI and DM revealed that the optimum lag for the mean, minimum and maximum temperature was on Day 47 (*r =* 0.21), 71 (*r =* 0.17), and 1 (*r =* -0.24), respectively. The optimum lag for the mean RH was on Day 70 (*r =* -0.29), while for the minimum and maximum RH, it was on Day 91 (*r =* -0.27 and *r =* 0.25, respectively). For rainfall, the optimum lag was on Day 64 (*r =* 0.33). Fig 6 illustrates the correlation plot between the mosquito density variables and the daily microclimate of the *Ae*. *aegypti* at the DH.

On the other hand, the ACF and PACF of the AI of the *Ae*. *albopictus* with prior values showed that the optimum lag was on Day 7 (*r =* 0.68) and 8 (*r =* -0.63), respectively. Meanwhile, the cross-correlation analysis between the AI and DM of the *Ae*. *albopictus* revealed that the optimum lag for the mean, minimum, and maximum temperature was on Day 45 (*r =* 0.26), 43 (*r =* 0.26), and 58 (*r =* 0.22), respectively. The optimum lag for the mean, minimum and maximum RH was on Lag Day 26 (*r =* -0.34), 28 (*r =* -0.38), and 19 (*r =* 0.24), respectively. Finally, the optimum lag for rainfall was on Lag Day 70 (*r =* 0.22).

**Fig 7:**
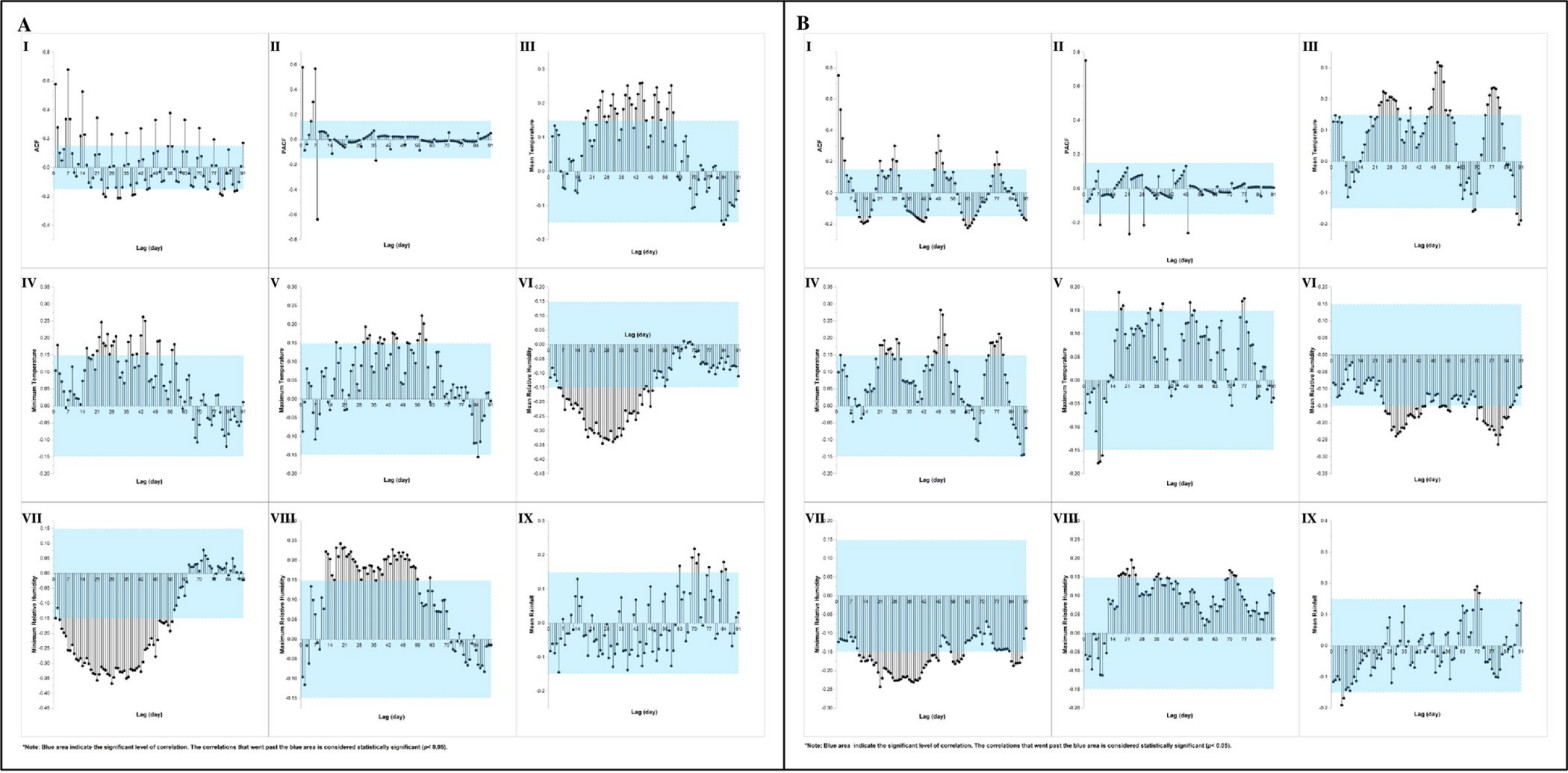
Autocorrelation (I and II) and cross-correlation (III to IX) of *Ae*. *albopictus* based on (A) adult index (AI), and (B) dengue positive trap index (DPTI) on daily microclimate at DH over 91 lag days. Note: Blue areas indicate significant levels of correlation.

Conversely, the autocorrelation analysis of the DPTI of the *Ae*. *albopictus* indicated that the optimum lag was on Day 1 (*r =* 0.75). The optimum lag for the mean, minimum, and maximum temperature was on Day 51 (*r =* 0.32), 50 (*r =* 0.28), and 17 (*r =* 0.19), respectively. Additionally, the optimum lag for the mean, minimum, and maximum RH was on Day 80 (*r =* - 0.26), 21 (*r =* - 0.24), and 23 (*r =* 0.20), respectively. The optimum TL for rainfall was achieved on Day 6 (*r =* 0.20). Fig 7 shows the correlation plots between the mosquito density and daily microclimate variables of the *Ae*. *albopictus* at the DH.

### Correlation between daily dengue cases, daily microclimate (DM), and Mosquito Indices (MIs)

**Fig 8:**
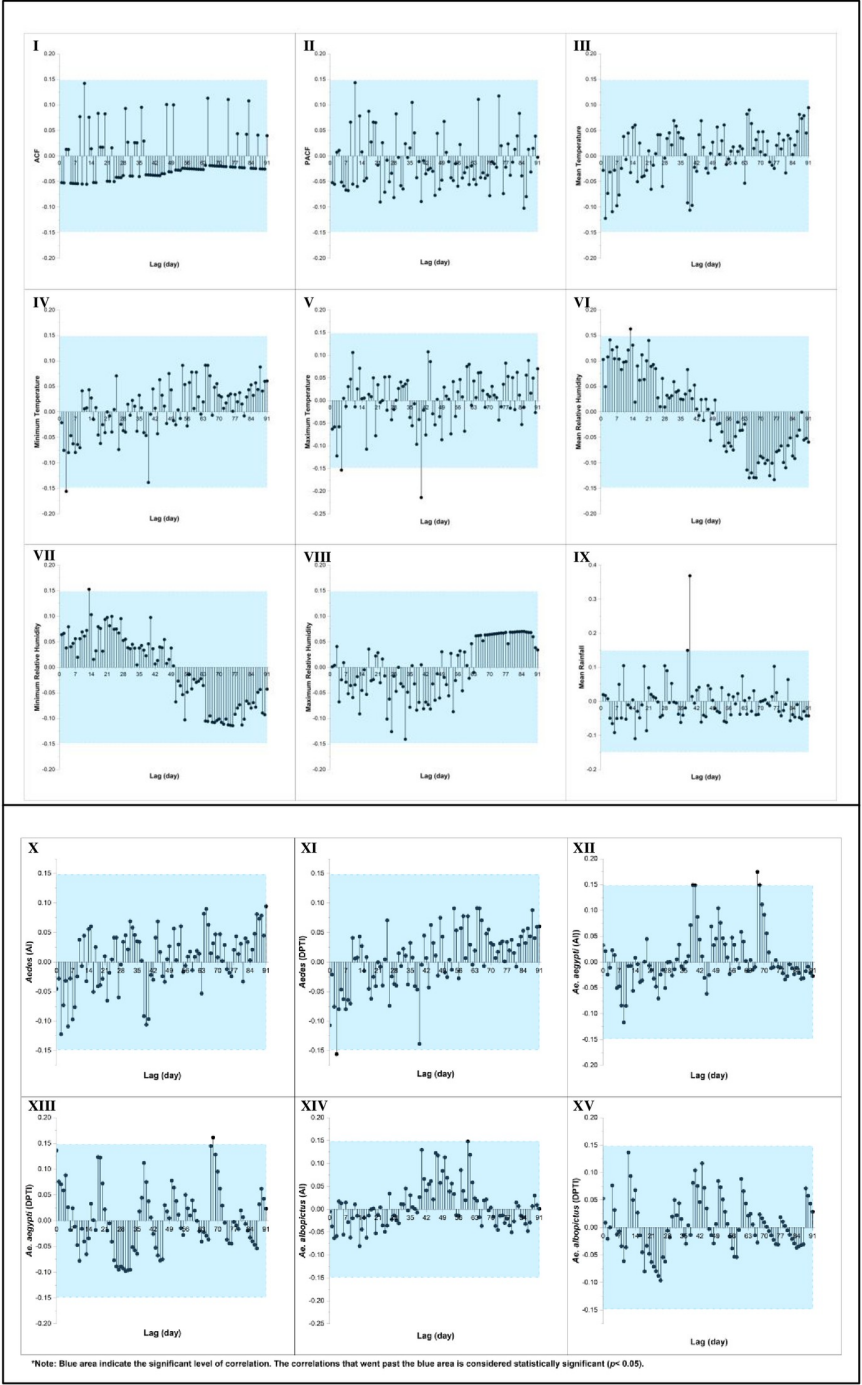
Autocorrelation (I and II) and cross-correlation (III to IX) between daily dengue cases and daily microclimate, and cross-correlation between daily dengue cases with mosquito density indices (X to XV) at the DH over 91 lag days.

The cross-correlation analysis between the daily dengue cases and daily microclimate in the DH revealed that the optimal TL for the minimum and maximum temperature was on Day 3 (*r =* -0.16) and 40 (*r =* -0.21), respectively. Additionally, the optimum lags for the mean (*r =* 0.16) and minimum RH (*r =* 0.15) were identified on Day 13, while the optimal lag for rainfall was on Day 39 (*r =* 0.37). Notably, no significant correlations were found between the AI and DPTI of the *Aedes* and *Ae*. *albopictus* and daily dengue cases (*p* > 0.05). However, the optimal lag between the daily dengue cases and the AI and DPTI of the *Ae*. *aegypti* was on Day 67 (*r =* 0.17) and 68 (*r =* 0.16), respectively. Fig 8 presents the correlation plots between the daily dengue cases, daily microclimate, and mosquito density at the DH.

## Discussion

In this study, dengue vectors were present at both the study sites, with only the *Ae*. *albopictus* at the NDH, whereas the *Ae*. *aegypti* and *Ae*. *albopictus* were coexisting at the DH. Even though the adult index at the NDH was high, the *Aedes* mosquitoes were more of a nuisance as no dengue virus was found in any of the *Ae*. *albopictus* samples collected. This was supported by the fact that there were no daily dengue cases at the site during the study period. Conversely, the dengue virus was detected in both *Aedes* species at the DH, with a slightly higher detection of the dengue virus in the *Ae*. *aegypti* despite the lower capture rate of the latter species compared to the *Ae*. *albopictus*. The dengue cases in DH were aligned with the detection of *Aedes* mosquitoes infected with the dengue virus at the site.

Meanwhile, the presence of both *Aedes* species at the DH was also consistent with other studies in Malaysia that described the *Ae*. *aegypti* and *Ae*. *albopictus* as sympatric species occupying similar ecological niches [54]. The probability was low that the *Ae*. *albopictus* also shared the same ovitraps with the *Ae*. *aegypti* as their oviposition sites. This finding indicated that the *Ae*. *albopictus* was highly flexible in its choice of oviposition sites for laying its eggs in natural as well as artificial containers in urban green areas and highly urbanised habitats with less vegetation cover [55,56].

Moreover, the overall results of the autocorrelation analysis of the AI (%) and DPTI (%) at both sites indicated that there was a positive correlation with the prior AI and DPTI, respectively. This might be due to the tropical climate in Malaysia, with its warm and humid weather and average temperature of approximately 20–32°C throughout the year [57]. The warmer climate in tropical areas, with its hot and humid weather and moderate rainfall, is ideal for mosquitoes to be active all year round. The adult index of all the mosquito species at the NDH and DH are positively correlated with the mean, minimum, and maximum temperatures at different time-lags. On the other hand, only the dengue positive trap index of the *Ae*. *albopictus* at the dengue hotspot area positively correlated with all the temperature variables, whereas the dengue positive trap index for the *Ae*. *aegypti* positively correlated with the mean and minimum temperatures but negatively correlated with the maximum temperature. A study on the correlation between the mosquito density variables captured using a BG-mosquito trap and meteorological factors in Quzhou City, China showed that the mosquito density of the *Ae*. *albopictus* positively correlated to the mean temperature and mean air pressure at a lag of 0–4 weeks [58]. Other study also showed similar results, where the mosquito density of the blood-seeking female *Ae*. *albopictus* in Arco and Riva del Gard, Italy was positively affected by the accumulated temperature over 3–4 weeks before sampling [25].

The different time lags for temperature in each mosquito species might have been due to the impact of climate propagation through the life stages instead of appearing immediately. Furthermore, each species has a different development time and life expectancy. One study has conducted on the duration of the development and life expectancy of wild strains of the *Ae*. *aegypti* and *Ae*. *albopictus* from Penang Island. Their findings showed that the duration of the immature stage of development for the *Ae*. *aegypti* and *Ae*. *albopictus* was 8.76 and 9.47 days, respectively. Meanwhile, the life expectancy for the *Ae*. *aegypti* was 19.94 days and the *Ae*. *albopictus* was 19.01 days [5]. The systematic review and meta-analysis conducted showed that the *Aedes* mosquito can survive at temperatures of 18–35°C, while the optimal temperature for dengue transmission is 29.3°C [23,36]. Additionally, other study showed that temperature extremes of 16 and 36°C significantly reduce adult longevity and female fertility [59]. The DPTI (%) was negatively correlated with the maximum temperature, which might be explained by the fact that the maximum temperatures at the NDH and DH (31.62 and 31.38°C, respectively) were higher than the optimal temperature for dengue transmission.

The AI and DPTI of *Ae*. *aegypti* and DPTI of *Ae*. *albopictus* at DH were negatively correlated to mean and minimum RH but has positive corelation to maximum RH. A low relative humidity can be mortal to an adult mosquito as it can cause the fluids inside the mosquito to evaporate through its spiracle [19]. A laboratory study implied that the oviposition of the female *Ae*. *aegypti* was inhibited and there was a reduction in egg fertility at a temperature of 35°C and RH of 60%. On the other hand, none of these effects were found in the *Ae*. *aegypti* at temperatures of 25–30°C and RH of 80% [16]. This was in line with the RH found at the non-dengue hotspot and dengue hotspot area, where both sites had a minimum RH of 53.59 and 30.36%, respectively, which were below the limit for the survival of the *Ae*. *aegypti*.

There was a positive correlation between the adult index and dengue positive trap index at both the study sites with rainfall more than 56 days later. Several studies support strong associations between accumulated rainfall and higher vector density more than four weeks later [25,60,61]. For example, a study on the effects of extreme climate on the *Ae*. *aegypti* in Kenya showed that the mosquito eggs and adults were significantly more abundant one month following an abnormally wet month [60]. Meanwhile, other indicated that the accumulated precipitation over 1–4 weeks before sampling negatively correlated with the mosquito density of the female host-seeking *Ae*. *albopictus* as events such as flooding and excessive rainfall can flush out breeding sites, thus reducing the vector population [62,63].

Lastly, there was very low to no correlation between the daily dengue cases, daily microclimate, and mosquito density in this study. This might have been because the daily dengue cases were underreported. In Malaysia, from 2014 onwards, the daily dengue cases are registered in the eDengue database only after the cases meet both the clinical case definition and laboratory confirmation of dengue fever [13]. Moreover, most asymptomatic dengue patients might not seek medical attention [64,65].

## Conclusion

The study conducted in both NDH and DH, where dengue interventions were implemented, revealed that the presence and high density of dengue vectors do not pose a threat when they are not carrying the dengue virus. Furthermore, the study identified the optimal lag times for dengue virus-infected and non-infected *Aedes* mosquitoes related to temperature, humidity, and rainfall. The different time-lags may be attributed to climate effects propagating through various life stages and different development times. However, the study is limited to two areas and may not fully capture the diversity of DH and NDH across the country. Moreover, there may be variability in environmental factors and mosquito abundance that are not accounted for in the analysis. Further study needs to be conducted include mosquito pesticide resistance and indoor capture rate before new interventions introduced to area with existing dengue interventions.

## Supporting information

S1 Table Rain gauge stations in Kuala Selangor and Petaling districts. (DOCX)
