# Supplementary material for: Temporal associations between microclimate, adult Aedes mosquito indices, and dengue cases at the residence level in Malaysia: Implications for targeted interventions
Source: PLoS One. 2025 Feb 3;20(2):e0316564. doi: 10.1371/journal.pone.0316564 (PMC11790129; doi:10.1371/journal.pone.0316564)
Supplement: S1 Table — (DOCX) [file pone.0316564.s001.docx]

**S1 Table: Rain gauge stations in Kuala Selangor and Petaling districts.**

| **No.** | **Kuala Selangor** | | | | **Petaling** | | | |
| --- | --- | --- | --- | --- | --- | --- | --- | --- |
|  | **Station ID** | **Station** | **Latitude** | **Longitude** | **Station ID** | **Station** | **Latitude** | **Longitude** |
| **1** | 3412001 | Sg. Tengi | 3.483 | 101.220 | 3115180 | Paya Jaras | 3.196 | 101.548 |
| **2** | 3411018 | Tg. Karang | 3.428 | 101.176 | 3115080 | Paya Jaras Hilir | 3.186 | 101.544 |
| **3** | 3411017 | JPS Tg. Karang | 3.423 | 101.171 | 3114087 | Subang Meru | 3.166 | 101.479 |
| **4** | 3411016 | Parit Sg. Burong | 3.455 | 101.147 | 220291 | Kg. Budiman | 3.155 | 101.472 |
| **5** | 3414032 | Rantau Panjang | 3.402 | 101.443 | 3017107 | S. Kembangan | 3.014 | 101.718 |
| **6** | 3313045 | Kg. Asahan | 3.363 | 101.361 | 3016077 | Jalan 222 | 3.096 | 101.634 |
| **7** | 3312047 | P/A Sg. Yu 1 | 3.363 | 101.239 | 3016102 | Kg. Gandhi | 3.076 | 101.621 |
| **8** | 3214056 | Sri Aman | 3.246 | 101.469 | 3015440 | Puchong Jaya | 3.042 | 101.621 |
| **9** | 3313070 | Parit Mahang | 3.297 | 101.378 | 3016100 | USJ 1 | 3.042 | 101.604 |
| **10** | 3313060 | Sg. Buloh | 3.308 | 101.321 | 3015001 | Puchong Drop | 3.019 | 101.597 |
| **11** | 3213057 | Braunston Jeram | 3.249 | 101.318 | 3115083 | Taman Mayang | 3.112 | 101.596 |
| **12** |  |  |  |  | 3115082 | Kota Damansara | 3.1567 | 101.577 |
| **13** |  |  |  |  | 3115084 | Kg. Melayu Sbg | 3.150 | 101.540 |
| **14** |  |  |  |  | 3015087 | Batu 3 | 3.075 | 101.553 |
| **15** |  |  |  |  | 3014096 | Taman Botani | 3.093 | 101.497 |
